# Economic status and catastrophic health expenditures in China in the last decade of health reform: a systematic review and meta-analysis

**DOI:** 10.1186/s12913-021-06408-1

**Published:** 2021-06-24

**Authors:** Qingqing Yuan, Yuxuan Wu, Furong Li, Min Yang, Dandi Chen, Kun Zou

**Affiliations:** 1 Department of Health Policy and Management, West China School of Public Health and West China Fourth Hospital, Sichuan University, Chengdu, Sichuan China; 2 West China Hospital, Sichuan University, Chengdu, Sichuan China; 3 West China Research Centre for Rural Health Development, West China School of Public Health, Sichuan University, Chengdu, Sichuan China

**Keywords:** Catastrophic health expenditure, Economic status, Systematic review, Meta-analysis, China

## Abstract

**Background:**

In order to solve the problem of “expensive medical treatment and difficult medical treatment” for patients and improve the equity of medical services, China started the health-care reform in 2009, and proposed ambitious goals of providing fair and high-quality basic medical and health services to all citizens and reducing economic burden of diseases. This study was to systematically explore the association between population economic status and incidence of catastrophic health expenditures (CHE) in mainland China in the last decade since 2009 health reform.

**Methods:**

This systematic review was reported according to the standard of preferred reporting items for systematic reviews and meta-analyses (PRISMA). We systematically searched Chinese Electronic literature Database of China Journal Full Text Database, Chinese Biomedical Journal Database, Wan fang Data Resource System, VIP Database, and English literature databases of PubMed, SCI, EMbase and Cochrane Library from January 2000 to June 2020, and references of included studies. Two reviewers independently selected all reports from 2000 to 2020 for empirical studies of CHE in mainland China, extracted data and evaluated the quality of the study. We conducted meta-analysis of the incidence of CHE and subgroup analysis according to the time of the study and the economic characteristics of residents.

**Results:**

Four thousand eight hundred seventy-four records were retrieved and eventually 47 studies with 151,911 participants were included. The quality scores of most of studies were beyond 4 points (91.49%). The pooled incidence of CHE of Chinese residents in the last two decades was 23.3% (95% CI: 21.1 to 25.6%). The CHE incidence increased from 2000 to 2017, then decreased over time from 2017 to 2020. From 2000 to 2020, the CHE incidence in rural areas was 25.0% (95% CI: 20.9 to 29.1%) compared to urban 20.9% (95% CI: 18.3 to 23.4%); the CHE incidence in eastern, central and western China was 25.0% (95% CI: 19.2 to 30.8%), 25.4% (95% CI: 18.4 to 32.3%), and 23.1% (95% CI: 17.9 to 28.2%), respectively; the CHE incidence was 30.9% (95% CI: 22.4 to 39.5%), 20.3% (95% CI: 17.0 to 23.6%), 19.9% (95% CI: 15.6 to 24.1%), and 23.7% (95% CI: 18.0 to 29.3%) in poverty group, low-income group, middle-income group, and high-income group, respectively.

**Conclusions:**

In the past two decade, the incidence of CHE in rural areas is higher than that of urban residents; higher in central areas than in eastern, western and other regions; in poverty households than in low-income, middle-income and high-income regions. Further measures should be taken to reduce the incidence of CHE in susceptible people.

**Supplementary Information:**

The online version contains supplementary material available at 10.1186/s12913-021-06408-1.

## Background

In 2009, China carried out the health-care reform, proposing the near-term goal of “effectively reducing residents’ burden of medical expenses and effectively alleviating ‘difficult and expensive to see a doctor’“, and the long-term goal of “establishing a sound basic medical and health system covering urban and rural residents, and providing the people with safe, effective, convenient, and inexpensive medical and health services”. Health-care reform has undergone major social and economic reforms, from pilot to comprehensive promotion, from industry reform to Healthy China strategy, from rapid population aging to major adjustments in family planning policies, which have brought fundamental changes to peoples’ lives and the society [1].

Since the implementation of the poverty alleviation policy, China has made great achievements. The number of poor people in the country had decreased from 165.67 million in 2010 to 30.46 million in 2017. The incidence of poverty decreased from 17. 2% in 2010 to 4.5% in 2016 [2]. However, among the current remaining poor people in China, poverty due to illness and return to poverty due to illness are the most prominent causes of poverty. Among the rural poor in my country, the proportion of poverty-stricken by disease and return to poverty due to illness reached 44.1% [3]. The incidence of disease has been one of the main causes of poverty, leaving patients and their families falling into short-term or long-term poverty [4].

CHE is a general term used to describe various health care expenditures that threaten households’ financial conditions to maintain their survival needs [5]. WHO defined CHE as health expenditures being more than 40% of household’s capacity to pay (CTP) [6, 7]. In the past decade, great achievements have been made in the National Health System Reform (NHSR) with significant improvement of access, quality and equity of health services, but there are also challenges that cannot be ignored, such as the roaring health-care costs. This is especially true in western China where the per capital income is far behind eastern China [8]. On the other side of the coin, improvement of access to health services, especially hospital services, may cause households to pay a large proportion of effective earnings, pushing households into financial hardship even poverty [9, 10].

Even though China has almost built up a universal health care system, which has played an important role in reducing the economic burden of residents’ diseases, the current basic medical security system is still sub-optimal to prevent residents’ CHE and poverty due to illness. The previous payment method of health expenditure in China was fee-for-service (FFS), which is one of the main reasons for the excessively high medical and health expenditure [11] . A study showed that in 2003, 2008 and 2013, 13.6, 15.1 and 13.8% of households experienced CHE, with a impoverishment rate of 8.6% due to disease, and 64% of non-survival expenditures for households actually incurring CHE, suggesting that CHE for the population had not improved or even increased [12], being heavy financial burden on the sick households and the society, and affected the health-care affordability of the population [13]. CHE is an important indicator of the economic incidence of disease, affecting the achievement of the “Universal Health Coverage” and the equity of the health financing system, as well as on social equity. What’s more, CHE is relative to each household, and there may be a significant imbalance between the income groups in terms of the degree of health financial security, the utilization of medical resources and the tolerance of disease incidence [14].

Therefore, the objective of this study was to examine the CHE in mainland China in the last two decades and its association with socioeconomic status of residents. And this research also explored the trends of CHE in the last two decades and gaps between regions.

## Methods

A systematic review was conducted according to the standard of preferred reporting items for systematic reviews and meta-analyses (PRISMA) [15].

### Literature search

We systematically searched Chinese literature databases including China Journal Full Text.

Database, Chinese Biomedical Journal Database, Wan fang Data Resource System, VIP Database, and English literature databases including PubMed, SCI, Embase and Cochrane library. The search terms included “catastrophic health expenditure,” “catastrophic medical expenditure,” “poverty-causing health expenditure,” “impoverishment,” “poverty-induced poverty,” “return to poverty due to illness,” “catastrophic health spending,” “catastrophic health spending,” “health payments,” “death by diseases,” “return to poverty due to illness.” The search strategy was firstly formed in PubMed and adopted for other databases (see appendix search strategy). In addition, reference lists of included studies were scanned for more eligible studies. The search period was from January 2000 to June 2020.

### Inclusion criteria

The inclusion criteria were: (1) research in mainland China; (2) quantitative studies including randomized controlled trial, non-randomized controlled trial, controlled before-after study, non-controlled before after study, cross-sectional study, cohort study or interrupted time series study; (3) the threshold for the incidence of CHE is 40%.; (4) all study populations; (5) and reported the incidence of CHE and economic status of participants. The language of literature was limited to Chinese and English, since almost all research in China were published in these two languages.

### Study selection

Firstly, two reviewers (Qingqing Yuan and Yuxuan Wu) screened the titles and abstracts of the identified citations and removed those obviously not relevant to the subject. Then, according to the inclusion criteria, two reviewers (Yuxuan Wu and Furong Li) select eligible studies by reading the full text. IF there was a disagreement, the three discussed until a consensus was reached. If consensus could not be reached, a senior researcher (Kun Zou orDandi Chen) was consulted for final decision.

### Data extraction and quality evaluation

Data Extraction and quality evaluation was performed by two reviewers independently (Qingqing Yuan, Yuxuan Wu). Different opinions were decided by discussion.

Data were collected using pre-designed data extraction table, including the author, year of publication, study location, study period, research design, characteristics of participants, sample size, number of CHE and economic status. If there were multiple reports on the harmonized data, priority is given to the most comprehensive publication. If two survey subjects come from the same survey data, select the literature with the most complete data to be included in the study. The quality of included studies was evaluated using the AHRQ scale [16]. The AHRQ had 11 entries, with an answer of “yes” with 1 point, and an answer of “no” or “not clear” with 0 point, with a total score of 0 to 11.

### Statistical analysis

Firstly, heterogeneity between included studies was examined using *I*^*2*^, with *I*^*2*^ > 50% indicated substantial heterogeneity. The pooled incidence of CHE was estimated with 95% confidence interval (CI) using fixed effect model if there was no heterogeneity (*I*^*2*^ < 50%). Otherwise, random effect model was used. Subgroup analysis was conducted by study period (three-years interval from 2006 to 2020), residency (urban or rural areas), the level of socio-economic development (categorized into east, central, and western regions, if the object of the survey area has multiple cities, then divided into other. The level of social and economic development in the east is the best, in the middle is general, and in the west is poor) [17], family income levels (poverty, low-income levels, middle-income, high-income, the poverty standard is based on the poverty line or the minimum living security line stipulated by the local civil affairs department, then divide the remaining families into three equal groups according to their income) [18], to explore their relationships with CHE. We also compared the growth relationship between the incidence of CHE and total health expenditure per capita (data from the National Bureau of Statistics of China) in different time periods. Meta-regression was performed to the disease type, the secular trend, the rural and urban residency, the social development level by region and the household income level adjusted for each other. All statistical analysis was conducted used Stata 15.0 software.

## Results

### Study selection

**Fig. 1:**
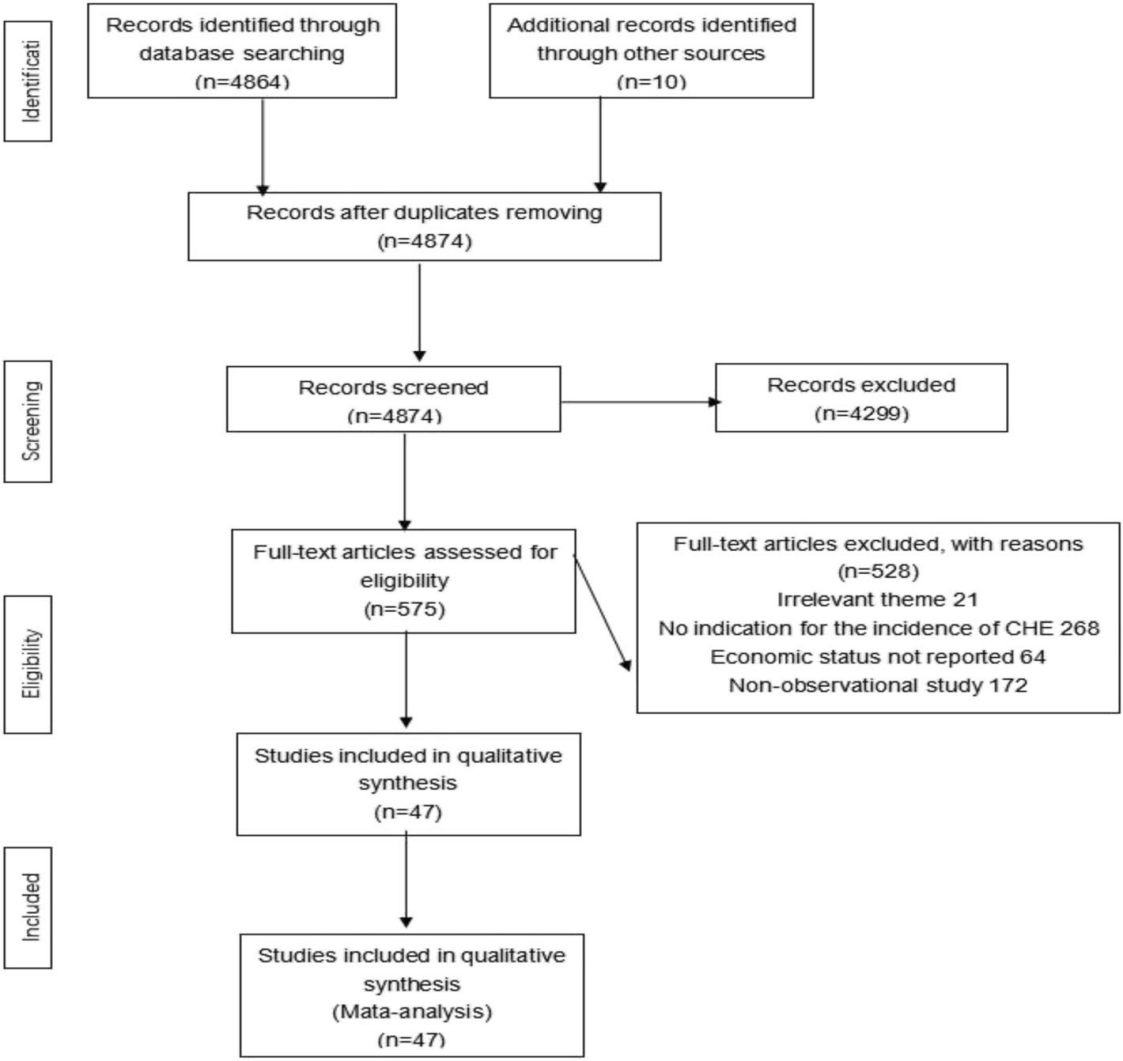
PRISMA flowchart of study selection

A total of 4874 citations were retrieved, 575 were likely to be relevant after viewing the titles and abstracts. Then by reading the full texts, 47 eligible studies (44 in Chinese and 3 in English) with 151,911 participants were finally included. (Fig. 1).

### Characteristics of included studies

A total of 47 cross-sectional studies including 151,911 participants were included in this study. The locations of studies were across-provinces (17), Yunnan (6), Hubei (4), Shandong, (4), Shanghai (3), Sichuan (3), Hebei (3), Shaanxi (2), Gansu (2), Jiangsu (2),Chongqing (2), Shanxi (2), Guangdong (1), Heilongjiang (1), Jiangxi (1), Liaoning (1), Qinghai (1), and Xingjiang (1). The included participants were general population (40), middle-aged and elderly people (4), rural villagers (30), cancer patients (2), HBV patients (2), etc. The quality scores of most of studies were between 4 and 9 points (81.67%) (Table 1).

**Table 1:** Characteristics of included studies

First author, year	Study location	study period	Study design	Study participants	Sample size	Incidence of CHE	Quality assessment of included studies
He 2006 [19]	Shanxi	2006	cross-sectional study	Families of farmers in poor counties	607	13.34%	5
Jia 2006 [20]	Shanghai	2006	cross-sectional study	Rural residents	3494	5.20%	3
Sun 2008 [21]	Qinghai	2008	cross-sectional study	Poor families (low-insured households) and non-poor families	1605	38.44%	5
Cui 2011 [22]	China	2011	cross-sectional study	Rural patients with primary hypertension, stroke or coronary heart disease	1189	19.20%	5
Wan 2011 [23]	Sichuan	2011	cross-sectional study	Low-income families	1980	19.90%	5
Chen 2012 [24]	Shanghai	2012	cross-sectional study	Active tuberculosis patients	97	34.02%	4
Chen 2012 [25]	Shandong	2012	cross-sectional study	Rural residents	179	26.51%	8
Yan 2012 [12]	Shaanxi	2012	cross-sectional study	Urban residents	800	5.38%	6
Ye 2012 [26]	China	2012	cross-sectional study	Urban residents	55,556	13%	10
Wang 2012 [27]	Hebei	2012	cross-sectional study	Town family	305	8.90%	5
He 2013 [28]	China	2013	cross-sectional study	Residents	2931	13.99%	4
Li 2013 [29]	China	2013	cross-sectional study	Residents	11,577	15.40%	7
Qin 2013 [30]	China	2013	cross-sectional study	Urban residents	4915	16.89%	5
Shi 2013 [31]	Yunnan	2013	cross-sectional study	Rural residents	300	4.67%	4
Ye 2013 [32]	China	2013	cross-sectional study	Families of maternal deaths in rural areas	195	56.92%	4
Fu 2014 [33]	Heilongjiang	2014	cross-sectional study	Residents	3098	23.79%	3
Liu 2014 [34]	Jiangsu	2014	cross-sectional study	Rural residents	1960	3.00%	6
Su 2014 [35]	Shandong	2014	cross-sectional study	Rural residents	2180	22.10%	4
Xu 2014 [36]	Guangdong	2014	cross-sectional study	Patients with stroke	300	35.33%	5
Yin 2014 [37]	Yunnan	2014	cross-sectional study	Rural residents	1140	11.19%	5
Li 2015 [38]	Xinjiang	2015	cross-sectional study	Residents	1845	6.97%	5
Che 2016 [39]	Yunnan	2016	cross-sectional study	Patients with chronic HBV without complications and indemnifying HBV cirrhosis over 20 years of age	940	41.70%	6
Gao 2016 [40]	Hubei	2016	cross-sectional study	Rural families	7202	9.71%	5
Li 2016 [41]	Chongqing	2016	cross-sectional study	Rural chronic disease residents	921	18.46%	5
Luo 2016 [42]	Hubei	2016	cross-sectional study	Rural residents	441	31.30%	6
Wang 2016 [43]	Hebei	2016	cross-sectional study	Rural residents	1581	10.37%	5
Wang 2016 [44]	Jiangsu	2016	cross-sectional study	Elderly residents in rural areas	1588	27.13%	5
Wu 2016 [45]	Jiangxi	2016	cross-sectional study	Lung cancer patients	203	47.30%	7
Xiao 2016 [46]	Chongqing	2016	cross-sectional study	People with high blood pressure in rural areas	1200	27.92%	5
Zang 2016 [47]	Gansu	2016	cross-sectional study	Urban residents	7500	15.40%	8
Zhang 2016 [48]	China	2016	cross-sectional study	Rural families	3012	29.71%	5
Zhou 2016 [49]	Sichuan	2016	cross-sectional study	Rural families	2244	6.37%	6
Feng 2017 [50]	Hubei	2017	cross-sectional study	Residents	604	26.99%	4
Gao 2017 [51]	China	2017	cross-sectional study	Patients with the disease	497	73.44%	3
Li 2017 [52]	Shanxi	2017	cross-sectional study	Urban residents	455	13.41%	5
Li 2017 [53]	Gansu	2017	cross-sectional study	Rural residents	373	31.09%	4
Niu 2017 [54]	Shandong	2017	cross-sectional study	Diabetes patients	2183	13.70%	5
Yu 2017 [55]	Shaanxi	2017	cross-sectional study	Farmers	250	76.80%	4
Duan 2018 [56]	Hebei	2018	cross-sectional study	Rural residents	1083	35.84%	3
Huang 2018 [57]	Yunnan	2018	cross-sectional study	Elderly diabetics	162	26.37%	6
Huang 2018 [58]	Shandong	2018	cross-sectional study	Rural families	754	19.73%	6
Li 2018 [59]	Yunnan	2018	cross-sectional study	Urban residents	1510	30.46%	5
Li 2018 [60]	Jiangsu	2018	cross-sectional study	Group of middle-aged and elderly people	1080	31.20%	4
Wang 2018 [61]	Sichuan	2018	cross-sectional study	Rural families	999	18.02%	9
Zeng 2018 [62]	China	2018	cross-sectional study	Urban residents	13,602	12.47%	5
Zheng 2018 [63]	Liaoning	2018	cross-sectional study	Cancer patients	1344	42.78%	6
Yu 2019 [64]	Yunnan	2019	cross-sectional study	Urban residents	3930	14.02%	8

### Pooled incidence of catastrophic health expenditure for Chinese residents

**Fig. 2:**
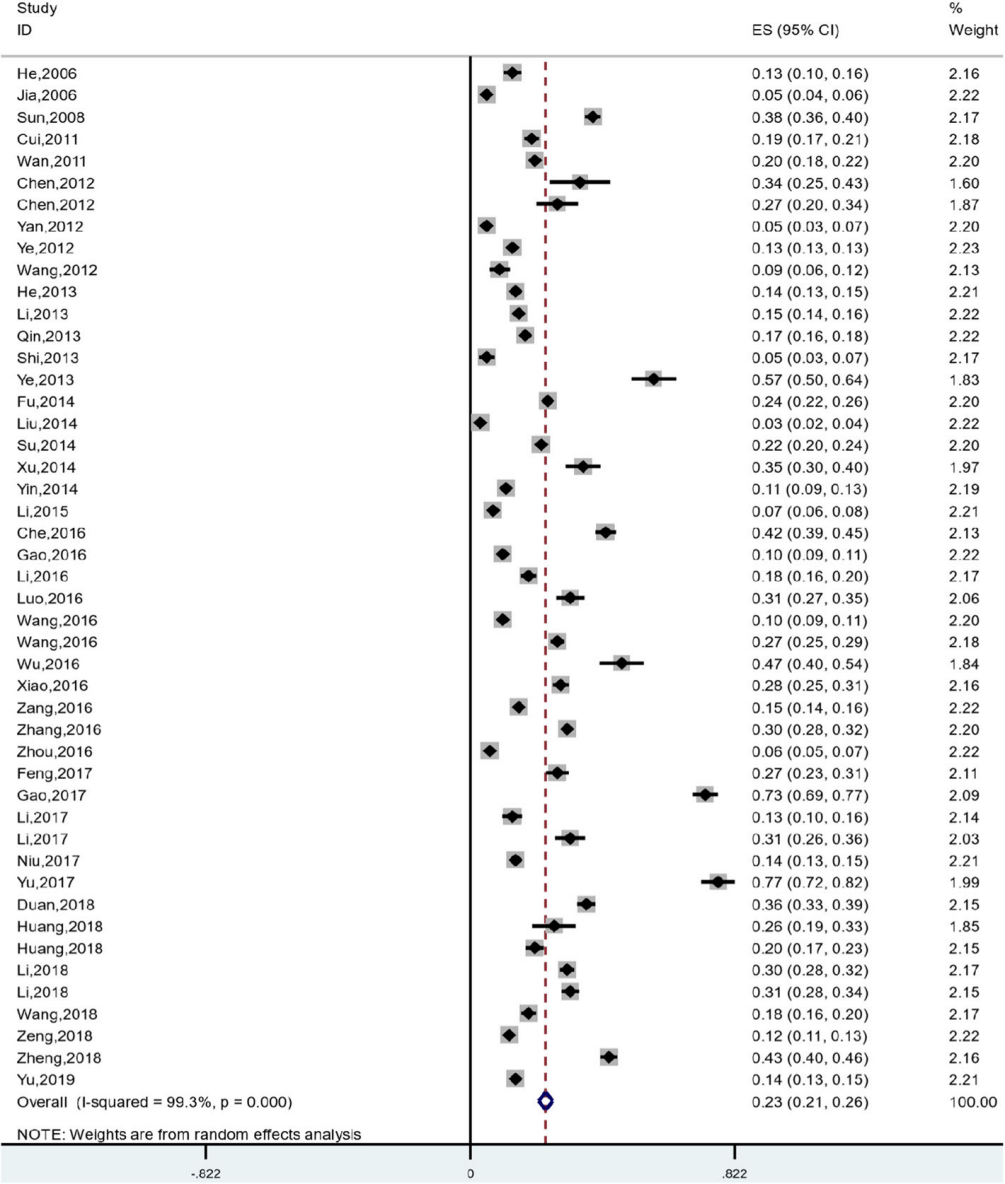
Meta-analysis of the combined incidence of catastrophic health expenditures for Chinese residents

The overall incidence of CHE in the past 14 years (2006 to 2020) among Chinese residents was 23.3% (95% CI: 21.1 to 25.6%), and there was a significant heterogeneity between the studies (*I*^*2*^ *= 99.3%*, *P < 0.001*), so random effect model was used (Fig. 2).

1

### Subgroup analysis

#### Secular trend

**Fig. 3:**
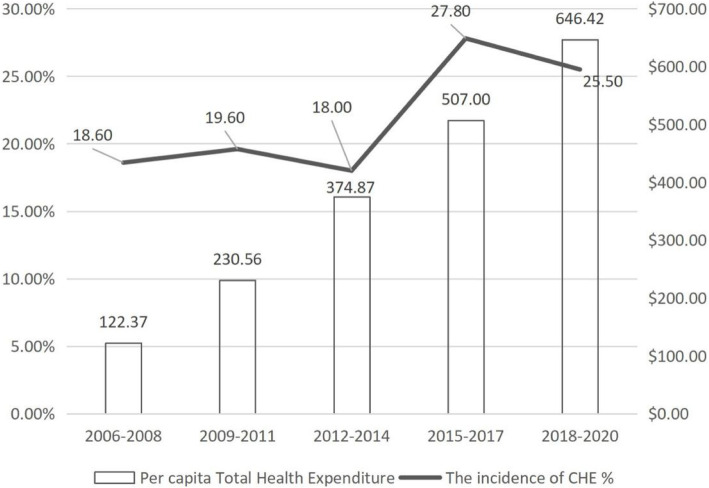
The time trends of catastrophic health expenditures and per capital totatl health expenditure for Chinese residents

In general, with the change of time, the incidence of CHE showed an upward trend. It was 18.6% (95%CI: − 1.5 to 38.8%), 19.6% (95%CI:18.2 to 21.0%), 18.0% (95%CI:14.9 to 21.0%), 27.8% (95%CI:22.6 to 33.0%), and 25.5% (95%CI:18.8 to 32.2%) for year 2006 to 2008, 2009 to 2011, 2012 to 2014, 2017 to 2017, and 2018 to 2020 respectively (Fig. 3, Additional file 1: figure 1 for forest map).

#### Rural and urban residency

The incidence of CHE in rural area was 25.0% (95%CI: 20.9 to 29.1%), higher than the urban 20.9% (95%CI:18.3 to 23.4%) (Additional file 1: figure 2 for forest map).

#### Social development level by region

The incidence of CHE in the central was higher than in the eastern, west region. The pooled incidence of CHE was 25.4% (95%CI: 18.4 to 32.3%) in the central, 25.0% (95%CI: 19.2 to 30.8%) in eastern China, 23.1% (95%CI: 17.9 to 28.2%) in the west and 18.8% (95%CI: 16.5 to 21.2%) in the rest (Additional file 1: figure 3 for forest map).

#### Household income level

The incidence of CHE in poverty group is higher than that of low-income group, middle-income group and high-income group. The incidence of CHE in poverty households was 30.9% (95%CI:22.4 to 39.5%), For the low-income, middle-income and high-income households, the CHE incidence was 20.3% (95% CI: 17.0 to 23.6%), 19.9% (95% CI: 15.6 to 24.1%), and 23.7% (95% CI: 18.0 to 29.3%), respectively (Additional file 1: figure 4 for forest map).

#### Meta-regression of associated factors of incidence of catastrophic health expenditure

In all studies, a meta-regression analysis was conducted to explore the impact of heterogeneous sources (including disease type, secular trend, rural and urban residency, social development level by region and household income level). The results show the disease type (*p* > 0.05), the secular trend (*p* > 0.05), the rural and urban residency (*p* > 0.05) and the social development level by region (*p* > 0.05) and the household income level (*p* > 0.05) are non-significant regulators (Table 2).

**Table 2:** The time trends of catastrophic health expenditures for Chinese residents

Variable	Coefficient	[95% Conf. Interval]		P
Other disease	0.186	− 0.773	1.144	0.696
Chronic disease	−0.114	− 0.776	0.547	0.728
Cancer	0.328	−0.956	1.612	0.606
2009 ~ 2011	0.471	−1.123	2.064	0.551
2012 ~ 2014	0.267	−0.825	1.359	0622
2015 ~ 2017	0.644	−0.422	1.709	0.227
2018 ~ 2020	0.747	−0.538	2.033	0.245
Rural	0.249	−0.522	0.572	0.927
Central	0.089	−0.956	1.135	0.863
East	0.062	−0.700	0.824	0.870
West	−0.166	−0.952	0.620	0.670
Low-income	0.021	−0.912	0.953	0.965
Middle-income	−0.224	−0.977	0.529	0.548
Poverty	0.395	−0.344	1.135	0.284
Cons	−2.199	−3.360	−0.793	0.003

#### Publication bias

There was statistically significant publication bias in included studies (*p*<0.001).

The funnel plot of publication bias was shown in Additional file 1: Figure 5.

## Discussion

There are four main findings in this study. First, the incidence of CHE in rural areas was significantly higher than that in urban areas. Second, the incidence of CHE in central areas was higher than that in eastern and western areas, and lowest in the other areas. Third, poverty group had higher CHE incidence than that of low-income group, middle-income group and high-income group. Finally, disease type may affect the incidence of CHE.

The incidence of CHE in rural areas was significantly higher than that in urban areas. The income gap between urban and rural areas makes the proportion of rural households’ cash health expenditure higher than that of urban households, and the rural population faces greater CHE risks. Although the establishment and expansion of the New Cooperative Medical Scheme (NCMS) reduces the medical burden of poor rural residents [65], the NCMS is lower than the Urban Employee Basic Medical.

Insurance (UEBMI) in terms of fund-raising, security level and reimbursement scope. The combination of the two causes a higher risk of CHE in rural households. In addition, from 2009 to 2014, the average cost of inpatients nationwide increased from 5951.80¥ to 8290.50¥, an average annual increase of 6.86% [11]. The excessively rapid increase in medical costs partially offset the protective effect of the basic medical insurance system. The burden continues to increase.

The incidence of CHE in central areas was higher than that in eastern and western areas. The results of Li′s research found that the incidence of CHE is in the opposite direction to the regional economic level: that is, the incidence of CHE in the more economically developed areas is lower, the incidence rate in the eastern areas is the lowest, the central region is the second, and the western region is the highest [11]. This study is contrary to its results. The reason may be related to thecharacteristics of the research object, the representativeness of different literature samples is different.

The poverty group had the highest incidence of CHE, followed by the high-income group, followed by the low-income group, and the middle-income group had the lowest incidence of CHE. There are many reasons for this phenomenon. On the one hand, it may be passively over-utilized health services due to induced demand [66], that is,these families are more likely to accept more expensive drugs or over-checks provided by doctors, thereby increasing medical expenses [67]. This is a relatively common phenomenon in China; on the other hand, high-income groups may actively over-utilize health services [67], such as using higher-level nursing services or wards. This will also increase medical expenses and make families incur excessive cash health expenditures, thereby widening the overall disparity in CHE of middle-income groups. Of course, in the less developed areas of China, it may not be common for farmers to choose such special needs, but this cannot be ruled out. The poverty group will also be affected by induced demand, but due to economic reasons, its affordability for medical expenses (including the cost of induced demand) is limited.

When medical expenses exceed their ability to pay, poor families generally choose Abandon treatment [68]. It is also because of its low income that it cannot pay large amounts of health expenses, and the gap in catastrophic health expenditure may be reduced, but this also makes the use of normal health services for some poor people suppressed and its health level will be seriously affected.

Disease type may affect the incidence of CHE. Wang’s research shows that the risk of catastrophic health expenditures in families with chronically ill patients is generally higher than the average of the overall population, and there are inequalities based on income gaps [69].Wu’s research shows that there are inpatients at home, and the risk of catastrophic health expenditure is 2.5 times higher than that of the general population [70].

The overall incidence of CHE in the past 14 years (2006 to 2020) among Chinese residents was 23.3% (95% CI: 21.1 to 25.6%).Compared with other countries, the global incidence of CHE at the 10% threshold was estimated as 9.7% in 2000, 11.4% in 2005, and 11.7% in 2010. In 2010, the incidence of CHE in Asia was 3.1% [71]. In 2015, the incidence of CHE in South Korea was 2.4% [72], and in 2021, Iran’s survey results show that the incidence of CHE is 4.7% [73]. The possible reason for this situation is that this study did not distinguish patients from the general population, so it may overestimate the incidence of CHE.

Some specific measures targeting economic vulnerable groups are needed in order to reduce the incidence of CHE in China. First, policies are needed to increase the protection effects of the three types of social medical insurance, including scope and reimbursement ratio. While expanding the coverage of medical insurance in China, it needs deepen the coverage of medical insurance, such as increasing the actual compensation ratio of medical insurance;Expand the scope of medical insurance reimbursement and allow more expensive special-effect medicines and treatments to enter the medical insurance reimbursement catalog. Second, increase the scope and intensity of medical assistance and social assistance. Further strengthen the protection of the disadvantaged groups, chronic patients and other vulnerable groups, and give inclined support to the system. Establish medical assistance for severely and seriously ill patients to prevent them from becoming poor due to illness or becoming poor due to illness. Third, control the excessive growth of medical expenses.

## Conclusion

Ten years after the new health reform in China, economic vulnerable groups still have higher risk of CHE than other groups. More research is needed to identify these population groups. And future health policies are warranted to contain healthcare cost, strength health financing protection and reduce the CHE in China towards universal health coverage.

## Supplementary Information

**Additional file 1.**

## Data Availability

The datasets used and/or analysed during the current study available from the corresponding author on reasonable request.
