# Supplementary material for: Economic status and catastrophic health expenditures in China in the last decade of health reform: a systematic review and meta-analysis
Source: BMC Health Serv Res. 2021 Jun 24;21:600. doi: 10.1186/s12913-021-06408-1 (PMC8229446; doi:10.1186/s12913-021-06408-1)
Supplement: Supplementary file 1 — Additional file 1. [file 12913_2021_6408_MOESM1_ESM.pdf]

Economic Status and Catastrophic Health Expenditures in China in the last decade of health reform: a systematic review and meta-analysis

Appendix

search strategy

PubMed
1. Catastrophic health expenditure
2. Catastrophic medical expenses
3. Poverty-causing health expenditure
4. Poverty due to illness
5. Poverty due to illness
6. 1 or 2 or 3 or 4 or 5
7. China
8. 6 and 7

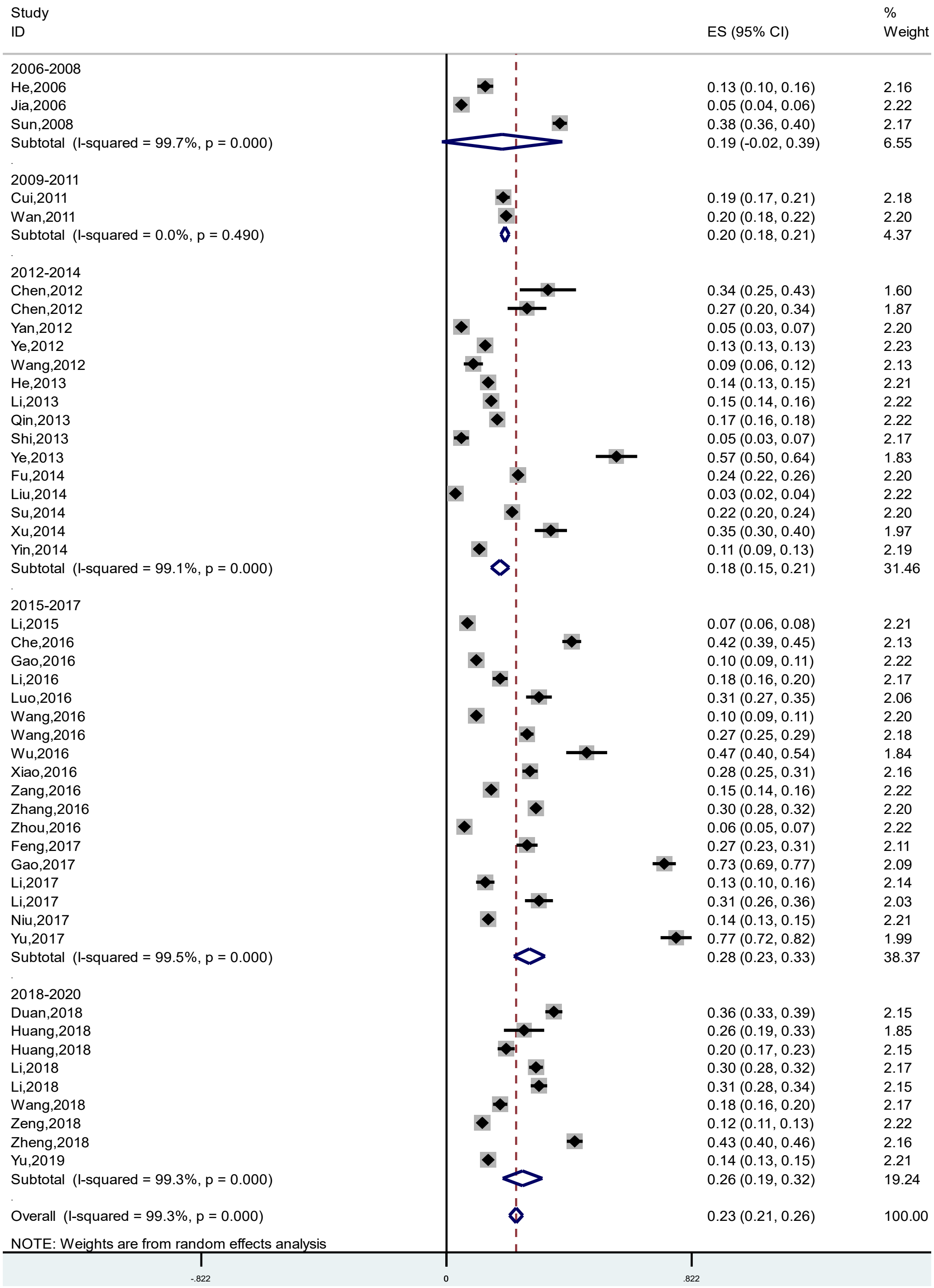

Figure 1 Meta-analysis of the time trends of CHE for Chinese residents

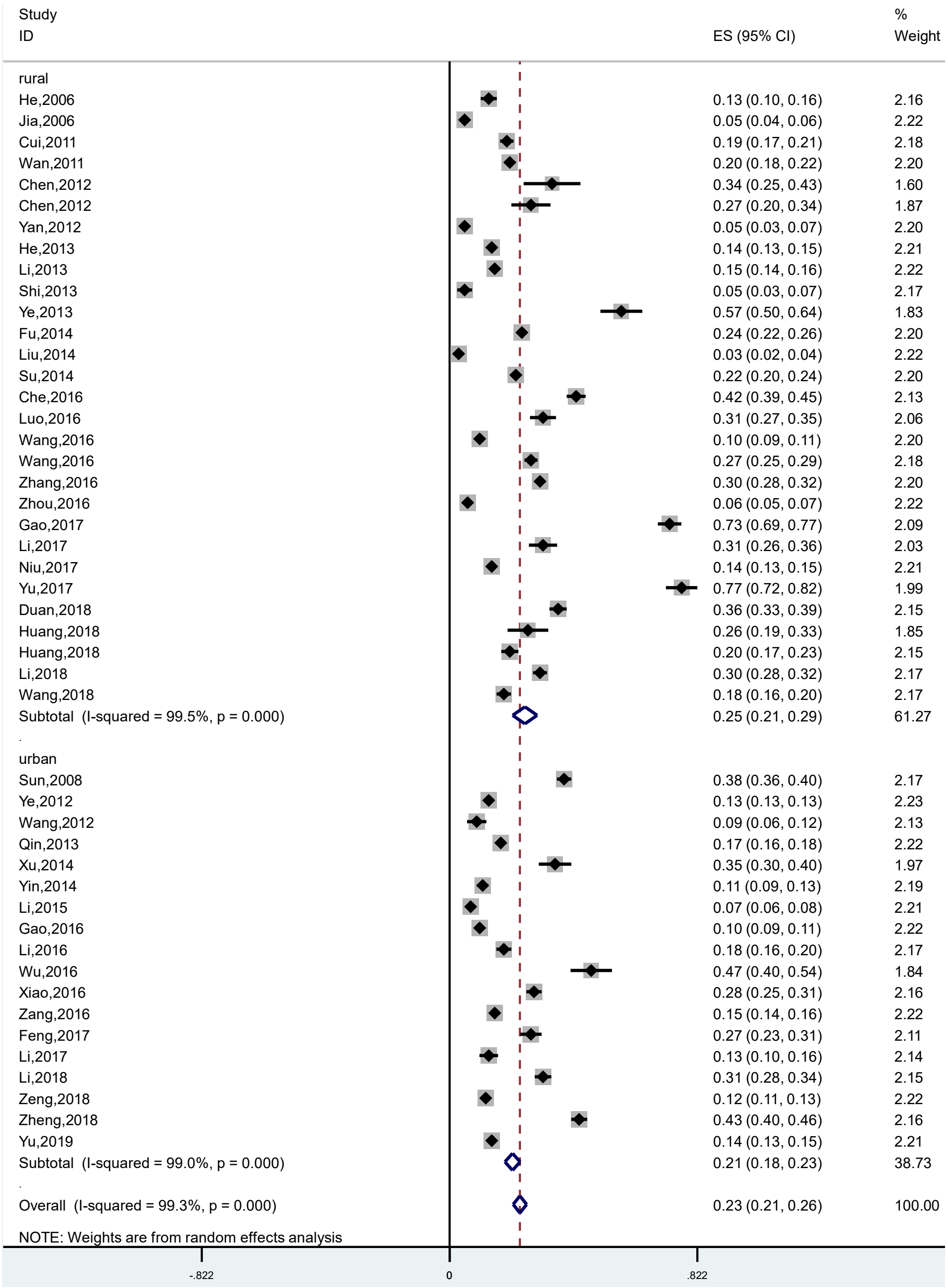

Figure 2 Meta-analysis of the incidence of CHE among rural and urban residents in China

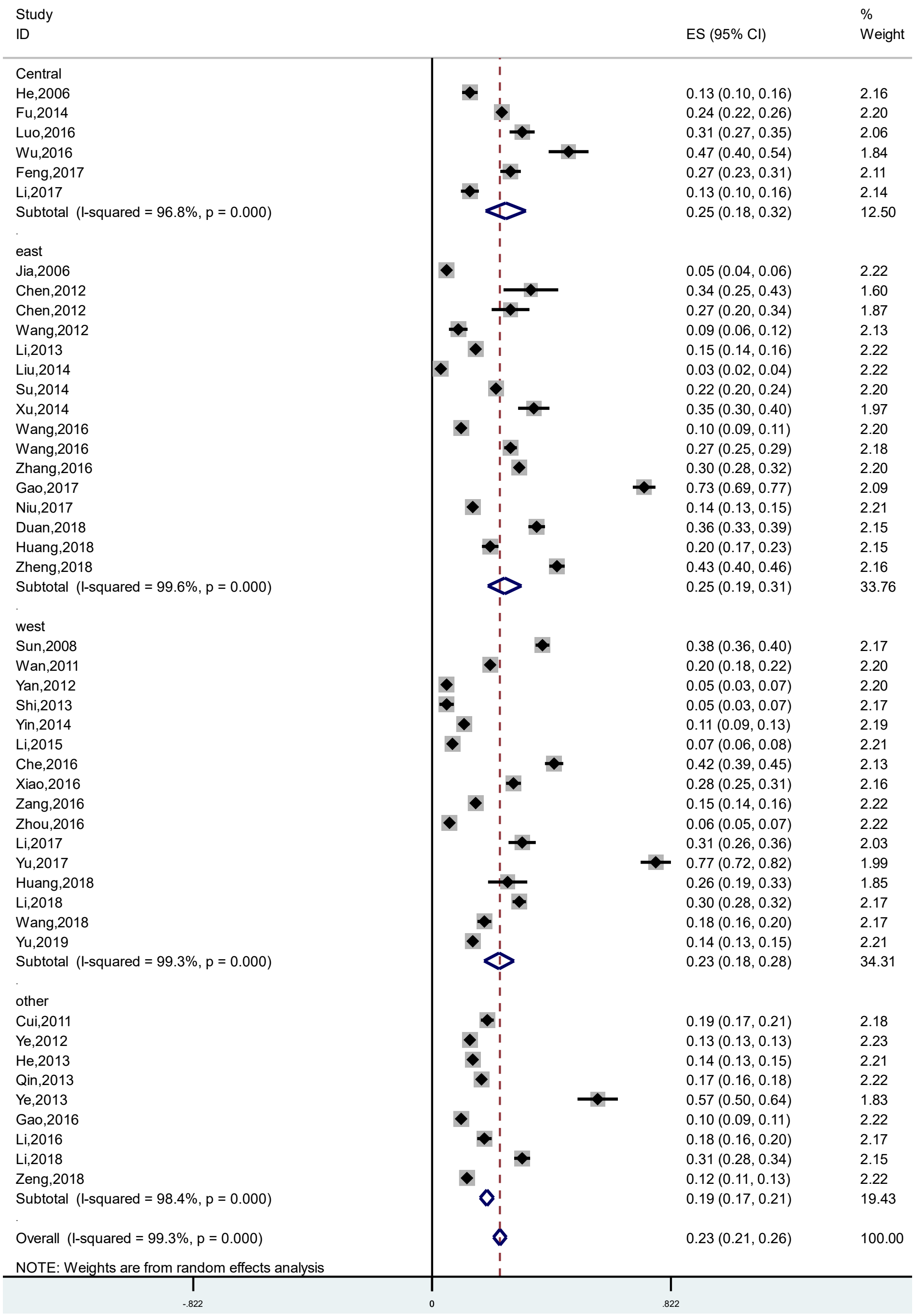

Figure3 Meta-analysis of the incidence of CHE at different levels of social development in China

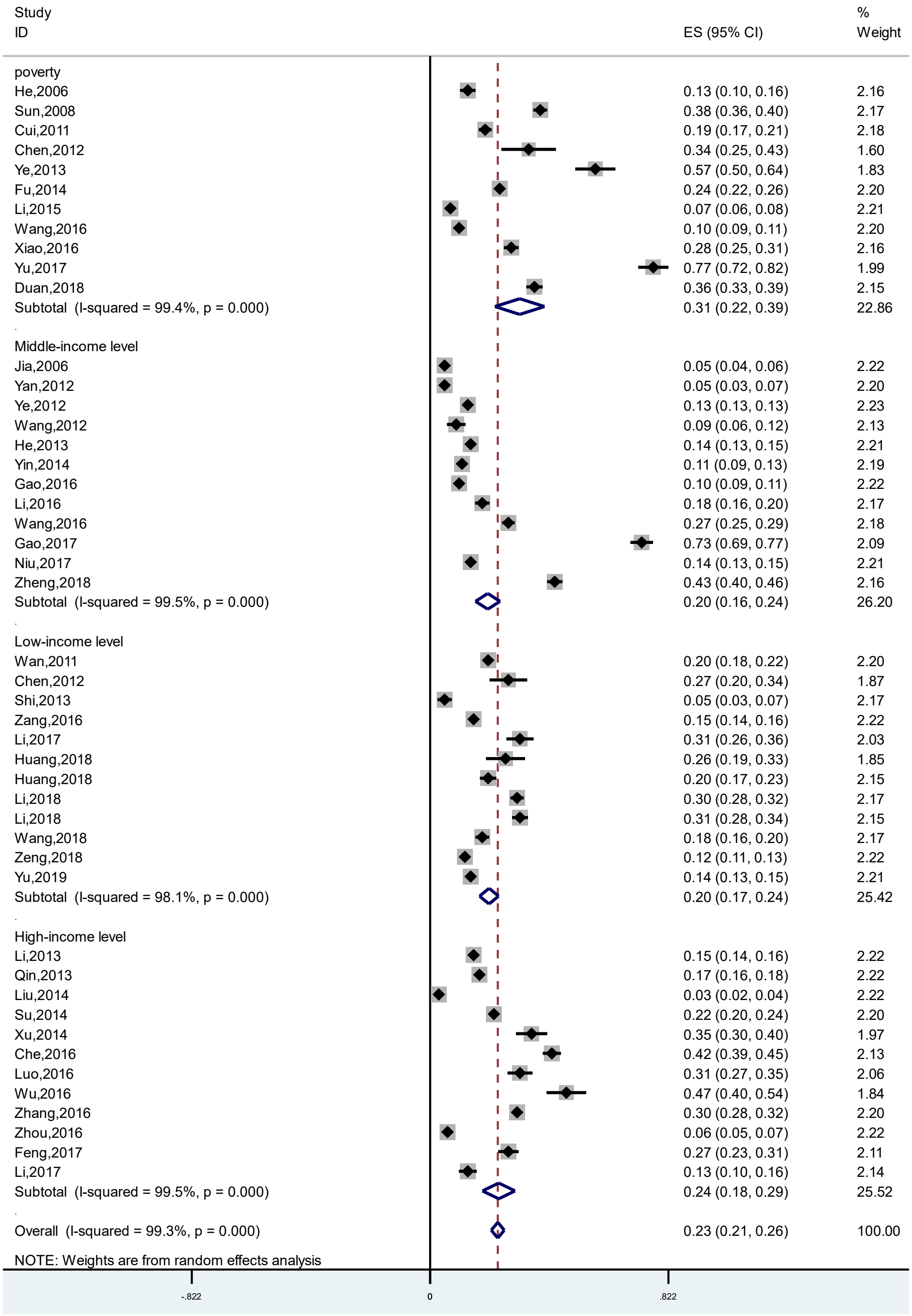

Figure 4 A meta-analysis of the incidence of CHE in different households in China

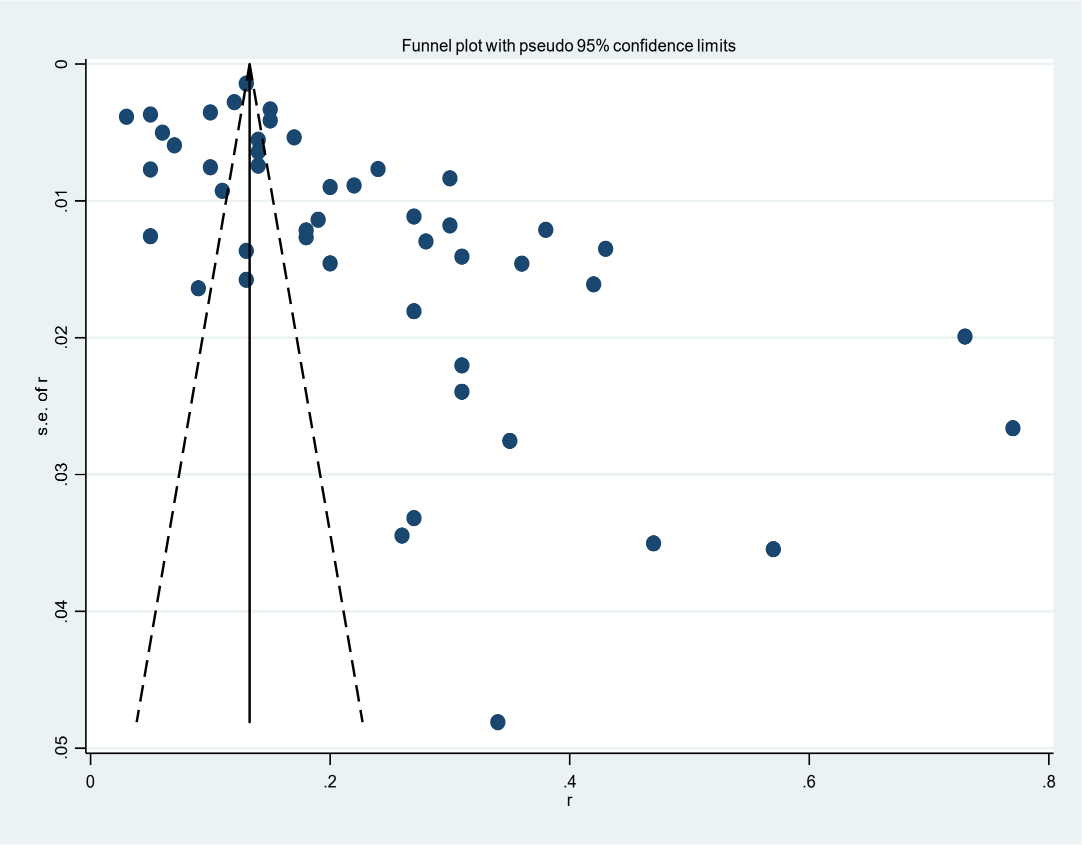

Figure 5 Publication bias
